# Study protocol for an efficacy trial of the “PrEP for Health” intervention to increase HIV PrEP use among people who inject drugs

**DOI:** 10.1186/s12889-023-15429-w

**Published:** 2023-03-17

**Authors:** Angela R. Bazzi, Michelle Bordeu, Katrina Baumgartner, Darien M. Sproesser, Christopher M. Bositis, Douglas S. Krakower, Matthew J. Mimiaga, Katie B. Biello

**Affiliations:** 1 Herbert Wertheim School of Public Health, University of California, 9500 Gilman Drive, San Diego, La Jolla, 92093 CA USA; 2 Department of Community Health Sciences, Boston University School of Public Health, Boston, MA USA; 3 Fenway Health, Boston, MA USA; 4 Greater Lawrence Family Health Center, Lawrence, MA USA; 5 Department of Family and Community Medicine, University of California, San Francisco, USA; 6 Beth Israel Deaconess Medical Center, Boston, MA USA; 7 Department of Epidemiology, Fielding School of Public Health, University of California, Los Angeles, USA; 8 Departments of Behavioral & Social Sciences and Epidemiology, Brown University School of Public Health, 121 South Main Street, Providence, 02912 RI USA; 9 Center for Health Promotion and Health Equity, Brown University, Providence, RI USA; 10 Herbert Wertheim School of Public Health, University of California, 9500 Gilman Drive, MTF 265E (Mail Code 0725), La Jolla, 92161 CA USA

**Keywords:** HIV infections, Opioid-related Disorders, Substance-related Disorders, Pre-Exposure Prophylaxis, Needle-Exchange Programs, Substance Use, Intravenous, Motivational interviewing, Self Efficacy, Patient Navigation, Social Cognitive Theory

## Abstract

**Background:**

HIV incidence has recently increased among people who inject drugs (PWID) across the United States, with outbreaks occurring in states with long-standing syringe service programs (SSPs) including Massachusetts (MA). Antiretroviral pre-exposure prophylaxis (PrEP) is an evidence-based HIV prevention strategy recommended for PWID, but uptake in this marginalized population is extraordinarily low.

**Methods:**

We describe the design and procedures for a National Institute on Drug Abuse (NIDA)-funded (R01) randomized controlled trial (RCT) testing the efficacy of “PrEP for Health,” a multicomponent behavioral intervention to increase PrEP uptake, adherence, and persistence among HIV-negative PWID attending SSPs in two areas of the U.S. Northeast that are heavily affected by injection-related HIV transmission. Participants are equally randomized to receive the “PrEP for Health” intervention (involving individually tailored HIV and PrEP education, motivational interviewing, problem-solving skills and planning, and ongoing navigation support) or an enhanced standard of care (eSOC) control condition involving a brief educational video on the utility of PrEP for HIV prevention. Co-primary outcomes are PrEP uptake (using medical/pharmacy records) and adherence (using tenofovir quantification in hair samples); a secondary outcome is PrEP persistence (using medical/pharmacy records) over 12 months. Major assessments occur at baseline, 1-, 3-, 6-, and 12-month follow-up visits. Planned analyses will examine intervention efficacy, specific hypothesized conceptual mediators of the intervention effect (e.g., self-perceived HIV risk; PrEP knowledge, interest in use, motivation, and behavioral skills) and epidemiologically linked moderators (e.g., age; gender; condomless vaginal or anal sex).

**Discussion:**

Findings from our extensive preliminary research with the study population revealed that a multicomponent, theory-based intervention targeting PrEP knowledge, motivation, self-efficacy, behavioral skills, and structural barriers to PrEP access is urgently needed for PWID who are at risk of HIV acquisition. We also learned that SSPs represent a highly acceptable service setting for delivering such interventions. In this study, we are evaluating the efficacy of the “PrEP for Health” intervention. If efficacious, findings from our implementation evaluation could help guide its dissemination to diverse SSPs and possibly other community-based settings accessed by this population.

**Trial registration:**

ClinicalTrials.gov number NCT04430257, registered June 12, 2020.

## Background

In the context of the ongoing opioid and polysubstance use crises in the United States, Human Immunodeficiency Virus (HIV) transmission among people who inject drugs (PWID) is a critical public health challenge [1, 2]. Increases in HIV incidence have recently been detected in this population after declining for decades, [3, 4] and concentrated HIV outbreaks among PWID have occurred in diverse U.S. regions, including areas with syringe service programs (SSPs) and other established HIV prevention services [5–19]. In Massachusetts (MA), a large HIV outbreak among PWID in two communities (Lawrence and Lowell, MA) from 2015 to 2018 appeared to be connected to increasing use of fentanyl (which increases injection frequency and receptive syringe sharing) and stimulants (which may increase sexual and injection-related exposures to HIV) [5, 7]. In addition to these behavioral factors, limited engagement in HIV prevention and treatment services due to low self-perceived HIV risk and structural factors that disrupt healthcare utilization (e.g., homelessness and incarceration) likely also contributed [5, 20]. Since 2018, additional clusters of incident HIV infections have been identified among PWID in Boston and other MA cities, [21] which may have worsened during the COVID-19 pandemic [22].

Antiretroviral pre-exposure prophylaxis (PrEP) is an evidence-based HIV prevention strategy that is recommended for PWID at risk of HIV acquisition, [23] yet PrEP research and actual uptake among PWID has been dismally low despite high potential for clinical benefit [24]. For example, in a survey of 423 PWID in the Greater Boston Area, 92% self-reported injection and sexual behaviors aligned with clinical indications for PrEP, but < 2% had ever used PrEP [25]. Our formative qualitative research in this region attributed low PrEP uptake among PWID to low self-perceived HIV risk, suboptimal PrEP knowledge and interest, [26] and multilevel barriers to accessing PrEP in clinical settings including pervasive stigma against PWID in healthcare systems [27, 28]. Nationally, PrEP prescribers’ limited willingness to prescribe PrEP to PWID may also limit uptake.[26, 29−31] While innovative low-barrier PrEP services have been implemented for PWID experiencing homelessness in Boston [32] and several other cities, [33, 34] evaluation research has highlighted significant challenges with PrEP adherence and persistence among PWID, [34, 35] underscoring the need for multicomponent behavioral interventions that target multiple hypothesized determinants of PrEP access and adherence [27, 36].

As PWID may have limited or ineffective interactions with traditional healthcare systems, [28, 37, 38] alternative, community-based and culturally-tailored settings may provide a more effective setting for PWID to access PrEP. Syringe service programs (SSPs), where staff routinely provide PWID with health information and support, [27, 28, 34, 37, 39−41] are highly acceptable venues for PrEP interventions for PWID, whether PrEP can be delivered onsite or through supported referrals to local, trusted clinics [34]. In our formative research, SSP staff in MA perceived PrEP to be aligned with their organizational missions and viewed PrEP integration into their existing services as acceptable and feasible [42]. Some SSPs already provide PrEP information and initial screening services (e.g., HIV testing), [43, 44] and SSP-based interventions that support adherence to medications for the treatment of Hepatitis C Virus (HCV) and opioid use disorder have been successful [45–47]. Given the growing numbers of SSPs nationally, [48] these venues have high potential for increasing PrEP uptake, adherence, and persistence among PWID.

Social Cognitive Theory (SCT) provides a valuable theoretical basis for behavioral interventions targeting the multilevel barriers to PrEP uptake, adherence, and persistence for PWID. SCT posits that people, their behaviors, and surrounding environments interact to influence how and when behaviors are performed, [49] with a focus on cognitions (i.e., knowledge), motivations, [50] self-efficacy, and behavioral skills and strategies for overcoming environmental (i.e., structural) barriers [51, 52]. SCT has proven useful in interventions supporting antiretroviral uptake and adherence (for HIV treatment and prevention) in at-risk, socially marginalized populations, including among those who use substances [53, 54]. Individually tailored education, motivational interviewing (MI), and problem-solving and planning are key SCT-informed strategies that could support PrEP uptake, adherence, and persistence among PWID if sufficient structural supports are also provided [55–58].

Patient navigation is an evidence-based, patient-centered healthcare delivery strategy that provides individually tailored supports to help people overcome barriers to accessing and staying engaged in healthcare services [59]. Patient navigation strategies have successfully improved HIV, HCV, and chronic disease care [59, 60]. Many of the activities included in these interventions (e.g., facilitating referrals and appointments, planning for transportation, [61] providing emotional support) could help address multilevel barriers to PrEP uptake among PWID, [26, 27] and patient navigators can also be trained to work successfully with marginalized populations (e.g., by avoiding stigmatizing language) [62–65]. As patient navigation alone may be insufficient in targeting all barriers to PrEP uptake and longer-term adherence and persistence, [66, 67] we hypothesized that a multicomponent intervention combining patient navigation with SCT-informed behavioral intervention strategies (individually tailored education, MI, problem-solving and planning) could effectively support PrEP uptake and develop a foundation of knowledge and skills upon which individuals’ longer-term PrEP adherence and persistence could be built [40, 68]. We thus developed the brief, multicomponent “PrEP for Health” behavioral intervention—delivered by trained PrEP patient navigators within SSPs—to improve PrEP uptake and adherence (co-primary outcomes) and persistence (secondary outcome) among PWID at risk of HIV acquisition. In this paper, following the Standard Protocol Items: Recommendations for Interventional Trials (SPIRIT) Statement [75], we detail the procedures for a randomized controlled trial (RCT) testing the efficacy of PrEP for Health within two SSPs in MA.

## Methods

### Overview of study design

**Fig. 1:**
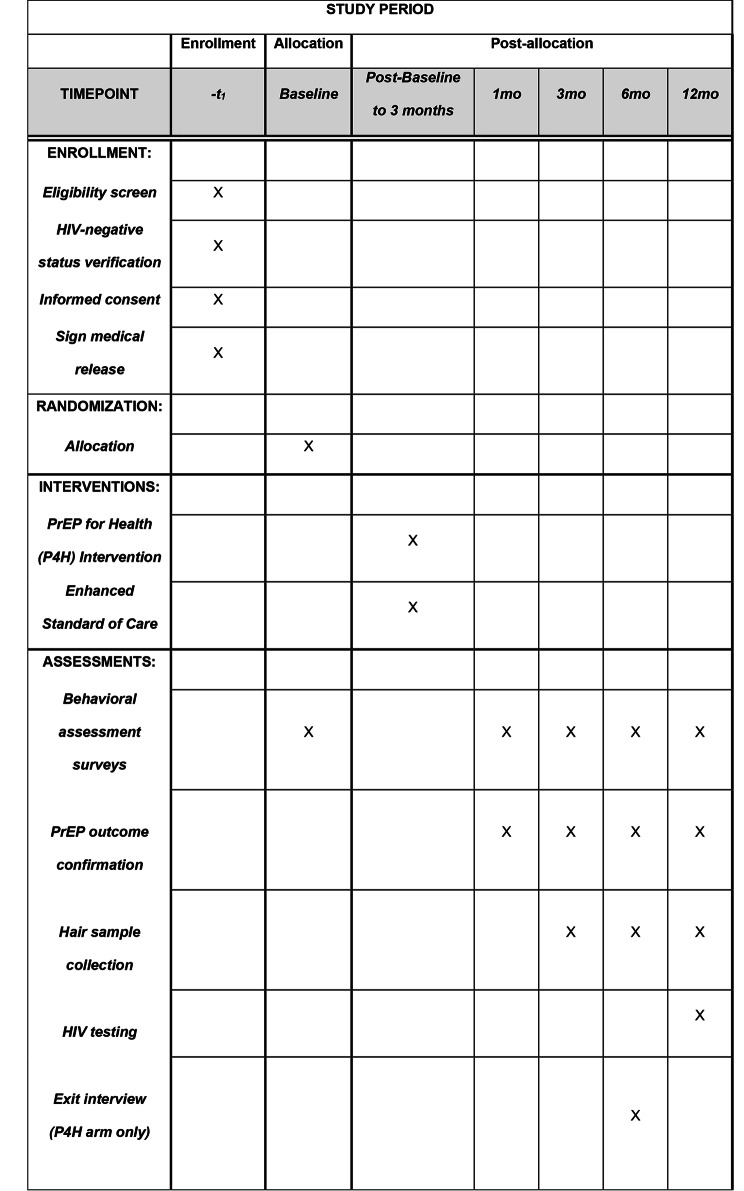
SPIRIT figure for the PrEP for Health trial

PrEP for Health is a two-arm RCT aiming to enroll 200 HIV-negative PWID attending two SSPs in MA (n = 100 per site). Based on the locations of recent HIV outbreaks and clusters among PWID, we partnered with two SSPs (i.e., study sites) in Lawrence and Boston/Cambridge, MA, jurisdictions heavily affected by opioid and stimulant use and injection-related HIV transmission. The SPIRIT figure of this trial, including the schedule of screening, enrollment and follow up visits, is illustrated in Fig. 1. In brief, participants are randomized 1:1 to receive the PrEP for Health intervention or an enhanced standard of care (eSOC) control condition, as detailed below. Participants are then followed for 12 months with major assessment visits scheduled at baseline and 1-, 3-, 6-, and 12-month follow-up timepoints. Participants receive escalating incentives across these assessment visits (ranging from $20-$50). Participants randomized to the PrEP for Health intervention also receive $10 for each of two primary intervention sessions. Participants provide written informed consent (described below). All study materials described in the sections below are available in English and Spanish (following professional translation and verification by bilingual research personnel). The Fenway Health institutional review board (IRB) reviewed and approved all study protocols. The following description of the protocol is based on Version 1.7, most recently updated and approved on 10/26/2022.

### Participant recruitment and screening

Participant recruitment involves active and passive methods. At each SSP, research team members (including research assistants, project manager, and navigators) and SSP personnel (including program managers and outreach specialists) approach potential participants seeking SSP services to briefly describe the study and assess interest in participating. At both study sites, screening is integrated into standard SSP intake processes to facilitate efficiency and reduce participant burden. Active recruitment also occurs when research staff accompany SSP personnel during wider community outreach (e.g., via mobile outreach to streets, parks, homeless encampments, shelters, and other local service organizations). Passive recruitment involves posting flyers and sharing business cards at the SSPs, nearby health centers, substance use treatment facilities, churches, and other places where PWID congregate. Recruitment is currently ongoing.

With support from SSP personnel, who often know potentially eligible members of the target population and can refer them to the study, research staff conduct brief eligibility screenings. Detailed eligibility criteria are described in Table 1. In brief, eligible individuals are ≥ 18 years old; self-report injection of drugs for non-medical reasons in the past month; and are HIV-uninfected (confirmed using a rapid HIV screening test or enzyme-linked immunosorbent assay; ELISA). Individuals may enroll in the study prior to confirmation of HIV serostatus but are withdrawn if test results indicate HIV-positive serostatus. Anyone receiving a positive HIV test result is referred to HIV care through standard processes at each SSP.

**Table 1:** Eligibility Criteria for the PrEP for Health trial

Inclusion	Exclusion
• Age 18 and older	• Does not understand and speak English or Spanish
• Any drug injection, past 30 days	• Unable to provide informed consent due to severe mental or physical illness, cognitive impairment, or substance intoxication at the time of enrollment
• Any HIV risk behavior, past 30 days ○ Receptive syringe sharing ○ Engagement in transactional sex ○ Any condomless sex	• HIV positive, confirmed by enzyme-linked immunosorbent assay (ELISA) testing already available at each SSP or by FDA-approved rapid HIV Antibody test if blood draws are unsuccessful
• Never used PrEP, or discontinued PrEP more than 6 months ago	

### Informed consent and enrollment

After confirming eligibility, trained study staff either conduct informed consent immediately or schedule an appointment for a later date, depending on participant preference. Study staff conduct informed consent in a private room using a consent document written according to Federal standards at or below an eighth-grade reading level that describes the study rationale, procedures, risks, benefits, measures to protect confidentiality, and participants’ rights and responsibilities. After explaining all study procedures, study staff then answer questions and gauge comprehension using a brief, structured assessment. Individuals who consent to participate sign the informed consent form, which is reviewed by the Site PI or their designees.

### Randomization

Following baseline assessments (see Fig. 1 and Measures section below for details), participants are randomized into either of the two study arms using a pre-programmed randomization module in REDCap. Randomization is site-specific to ensure balance within site. After randomization, participants randomized to the PrEP for Health intervention condition can elect to have their initial intervention session with the PrEP Navigator that day or schedule an appointment for a later date.

### Timing of assessments

Research staff administer assessments at baseline (pre-randomization) and 1-, 3-, 6-, and 12-month follow-up visits (post-randomization). Assessments are not blinded, however only research staff uninvolved in intervention delivery administer follow-up assessments to reduce social desirability bias. Moreover, study staff use standardized survey instruments and are instructed in how to mitigate bias in survey administration. Between 6- and 12-month assessments, check-ins (by phone or in-person) are incentivized ($10/check-in) to update locator information, support retention, and facilitate ongoing communication between major assessments.

### PrEP for Health Intervention condition

PrEP for Health is a two-session, manualized, multicomponent intervention grounded in SCT and informed by prior interventions with similar populations. Given that a key strategy is MI, draft manuals were reviewed and refined by a certified Motivational Interviewing Network of Trainers (MINT) trainer. The intervention is delivered by trained PrEP Navigators through up to two in-person intervention sessions at SSP study sites, followed by ongoing navigation support for a period of three months. The two in-person intervention sessions cover PrEP uptake (i.e., “Module 1,” which may be repeated during the second intervention session if necessary), and PrEP Adherence and Persistence (i.e., “Module 2”). Navigators are individuals without healthcare training who have personal or professional experience working with PWID (i.e., they are not licensed clinical social workers and no other certifications or licenses are required).

Interventionist training and supervision: PrEP Navigators receive extensive training in the intervention manual content (including detailed background information on HIV transmission and PrEP); MI (involving an initial 8 h of self-paced web-based training); principles of harm reduction (including overdose prevention), cultural humility, and trauma-informed services; problem-solving and de-escalation techniques; and research ethics. An MI-experienced licensed clinical social worker conducts ongoing supervision and training to enhance intervention delivery and ensure fidelity to the intervention manual and principles of MI.

Module 1: PrEP uptake: In the first in-person intervention session, PrEP Navigators use specific strategies to deliver individually tailored HIV and PrEP education and MI to address HIV risk perceptions and increase PrEP interest and motivation. PrEP Navigators also use this session and ongoing follow-up interactions (described below) to engage participants in problem-solving and planning to build their self-efficacy and behavioral skills to facilitate the process of obtaining initial PrEP screening and prescriptions. PrEP Navigators use patient navigation techniques to assess barriers to PrEP uptake, navigate the PrEP medical care system (e.g., through supported referrals to SSP-based or other local prescribers familiar with healthcare for PWID), and engage participants in problem-solving and planning around anticipated challenges in obtaining PrEP prescriptions (e.g., overcoming insurance or copay barriers). If participants do not initiate PrEP within one month of this initial intervention session, this PrEP uptake-focused content (i.e., Module 1) can be repeated in participants’ second intervention session to help trouble-shoot around initial uptake.

Module 2: PrEP adherence and persistence: For participants who initiate PrEP, in the second in-person intervention session, PrEP Navigators use similar strategies described above (e.g., MI, patient navigation techniques, problem-solving and planning) to motivate and support participants in achieving optimal PrEP adherence and persistence. This includes problem-solving around adherence challenges and anticipated adherence facilitators informed by evidence-based interventions and formative research (i.e., specific “steps” towards optimal adherence such as planning for transportation to pharmacies and PrEP care appointments). Finally, PrEP Navigators support participants in developing an individually tailored PrEP routine, including planning for future PrEP prescription refills and care appointments, and devising plans for handling missed doses and lapses in PrEP care.

Ongoing PrEP navigation support: For three months after the initial intervention session, PrEP Navigators provide additional, ongoing MI and PrEP navigation, as needed, through in-person, phone, and text-based interactions to support participants in coping with ongoing or new challenges with PrEP uptake, adherence, and persistence. Near the end of this three-month period, PrEP Navigators’ help participants plan for ongoing PrEP adherence and persistence.

### Enhanced standard of care control condition

Following randomization, participants in both the PrEP for Health intervention and eSOC control conditions watch a brief educational video on PrEP that was adapted by our research team from existing resources (e.g., whatisprep.org). The video is available in English and Spanish and uses non-technical terminology to describe what PrEP is and how it works to prevent HIV via sexual and injection exposures. Additionally, all participants, regardless of study condition, are eligible to receive any services offered at the local SSP study sites, which includes referrals to local PrEP clinicians and other healthcare providers. Moreover, participants who attend local PrEP clinics and are deemed clinically eligible for PrEP may be prescribed PrEP according to standard clinical practices.

### Measures

At all major assessment visits, participants complete interviewer-administered assessments of HIV and PrEP knowledge, perceived HIV risk, PrEP interest and self-efficacy, and patterns of substance use, sexual behaviors, physical and psychosocial health conditions, and health service utilization. Socio-demographics (e.g., race/ethnicity, age) are assessed at baseline only. Medical record review is conducted throughout the course of the study. Biological measures of adherence to oral PrEP (i.e., concentrations of tenofovir in hair samples [70, 71]) are collected at 3-, 6- and 12-month follow-up visits only, and incident HIV infection is assessed at 12-month follow-up visits only (Table 1). Because a long-acting injectable PrEP medication has recently been approved for prescribing among people who have sexual exposures to HIV, [71–74] some participants might use this newer formulation instead of oral PrEP medications. For participants who use injectable PrEP, which must be administered by a healthcare professional, we will track adherence using medical and pharmacy records, as biomarkers of adherence to injectable PrEP are not available and may not provide additional information on adherence beyond review of medical and pharmacy records.

Co-primary outcome measures: Our first co-primary outcome, PrEP uptake, is primarily assessed via visual confirmation that a PrEP prescription was filled and received. At each follow-up assessment, we also request that participants bring in their PrEP prescription packaging to confirm that the prescription was picked up. For injectable PrEP, uptake will be defined as receipt of their first injection,

Our second co-primary outcome, PrEP adherence, is assessed only among those who report initiating PrEP (i.e. taking at least one pill or receiving at least one injection). We assess adherence at 3-, 6-, and 12-month follow-up visits by collecting hair samples to test for the presence of detectable levels of tenofovir, which reflect adherence to either tenofovir disoproxil (TDF) or tenofovir alafenamide (TAF) over approximately the prior month. Samples are ~ 50–100, 1-2 mm-long strands of hair cut with scissors. Samples will be analyzed at the University of California, San Francisco, Hair Analysis Lab for quantification of tenofovir levels [69]. We supplement this biological monitoring with self-reported adherence at all major assessment visits. For injectable PrEP, which involves a monthly intragluteal injection for one month and then bimonthly injections thereafter, optimal adherence will be defined as receipt of injections within one month of their target injection date, which is the time at which people would need to reinitiate monthly injections for two injections (i.e., reload) due to waning drug levels.

Secondary outcome: We assess our secondary outcome, PrEP persistence, using pharmacy and medical records to confirm prescription refill maintenance and attendance at PrEP care follow-up visits. PrEP persistence will be defined as having ≥ 16 days of oral PrEP medication filled per 30-day period, or receiving PrEP injections within one month of their target dates, for at least three-quarters of the months since initiation to study completion (approximately 9/12 months) [76].

Conceptual mediators: Informed by our preliminary studies, conceptual mediators of intervention effects are measured by self-report at each major assessment visit and include HIV knowledge (assessed using selected and adapted items from the International AIDS Questionnaire [78] and the Marsch HIV/AIDS knowledge test [78, 79]), perceived HIV risk (using items adapted from the validated Perceived Risk of HIV Scale [80]), and PrEP knowledge, interest, motivation, and behavioral skills (using items adapted from research with people who use drugs [81]). PrEP use intentions and self-efficacy are measured using selected and adapted items from work by Walsh, [82] and anticipated PrEP stigma from work by Calabrese, et al. [83]. Structural barriers to PrEP access include healthcare access and utilization (items from the National HIV Behavioral Surveillance Survey [84]) and stigma from medical providers (from Fong, et al. [85]).

Epidemiologically linked moderators: Also based on our formative research, we hypothesize observing heterogeneity in intervention effects according to participant age, gender, and baseline sexual risk (specifically, condomless vaginal or anal sex) and polysubstance use (including stimulants), assessed by self-report at baseline and follow-ups assessment visits.

### Statistical analyses

Planned preliminary analyses include assessing distributions and autocorrelations across time for all variables. As we are utilizing interviewer-administered survey assessments, the primary anticipated reason for missing data is participant attrition. Based on our preliminary studies, we are anticipating and accounting for 20% attrition. Attrition effects will be evaluated by testing whether systematic differences exist between participants who complete the research study and those who discontinue participation. We will explore strategies (e.g., multiple imputation) for imputing missing data based on patterns of missingness, using sensitivity analyses to determine the optimal method of handling missingness. Dependent variables will be examined to determine which distributional models are most appropriate for subsequent statistical procedures. We will examine the equivalence of the random assignment with regards to key baseline characteristics, including socio-demographics and risk behaviors.

Aim 1: Our primary analysis will compare PrEP uptake and adherence (co-primary outcomes) between the PrEP for Health intervention and eSOC conditions. For PrEP uptake, we will perform chi-square tests to examine group differences in proportions over the course of the study, and Cox proportional hazard models to assess differences in time to PrEP initiation. For PrEP adherence, we will initially assess differences in adherence at 3-month follow-up visits. We will also conduct longitudinal analyses to assess differences in adherence over the course of the study (12 months). We will use generalized linear models (GLMs) with properly chosen link functions. GLMs will be estimated using generalized estimating equations with robust standard error estimates (GEE), which provide an extension of regression analysis to the case of correlated or repeated observations and allows for inclusion of both categorical (e.g., binomial) and count-dependent variables (e.g., Poisson, zero-inflated) and appropriate modeling of covariance structures when observations are correlated across time. We will follow an intent-to-treat model, analyzing participants according to the study arm to which they are assigned, regardless of fidelity to assigned group. Sensitivity analyses will compare those who receive the intervention session(s) to those who do not. All analyses will use two-tailed tests of significance, with significance at alpha level 0.05.

Aim 2: For mediation analyses, we will explore the role of hypothesized mediators using structural equation modeling (SEM) to determine whether the intervention impacts PrEP uptake and/or adherence through our hypothesized mediators. SEM will allow for the simultaneous estimation of total, direct, mediated, and indirect effects of the intervention on the outcome(s) via a set of multiple mediators, simultaneously. The fit of the overall model will be assessed, and individual paths will be assessed using path coefficients, standard errors for each path coefficient, and tests of significance of each coefficient [86]. Inferences for indirect effects will be estimated using bootstrapped confidence intervals.

Aim 3: To assess heterogeneity of intervention effects, we will examine interactions between the intervention and hypothesized moderators on both the multiplicative and additive scales. Multiplicative interaction will be estimated by including a treatment x moderator term in primary analyses described in Aim 1 above. Additive interaction will be estimated by calculating the Attributable Proportion due to Interaction (API), the proportion of the excess risk that is due to the presence of both the intervention and the moderator simultaneously. Significant or large interaction terms suggest that intervention effects differ by subgroups of the moderators. We will then perform stratified subgroup analyses to further investigate and describe heterogeneity between groups of individuals.

### Sample size calculations

The primary power analysis was based on differences between the PrEP for Health intervention and eSOC conditions in PrEP uptake (co-primary outcome). Effect sizes are estimated from our pilot studies, as well as results of a large mathematical modeling study examining PrEP use among U.S. PWID [87]. Assuming baseline uptake at 25%, with uptake of 50% in the PrEP for Health intervention arm (effect size: d = 0.60), we need 80 completers per condition to have 90% power to find a significant difference at alpha = 0.05. Assuming 20% attrition, we will need to enroll 200 PWID (100 per arm).

### Data management, safety and monitoring

All survey data is inputted directly into REDCap, a HIPAA-compliant, comprehensive data management system. Hard and soft-copy participant data is identified by an ID number only, and a link between names and ID numbers is kept separately in a password-protected file. Name-based files are stored separately from survey data. Soft copy data is stored on study-specific secure and password protected network drive folders and is only accessible to study staff. Hard copy data is stored in locked cabinets within restricted and secure areas at study sites.

All study staff are trained in confidentiality and have signed confidentiality agreements. Study staff have been trained in ethical human subject research practices to minimize participant risk. The investigators report unanticipated problems, safety monitors’ reports, and adverse events to the Fenway Health IRB in accordance with IRB policies.

Given that this is a behavioral intervention with minimal risk, the study has no stopping rules and interim analyses will not be conducted. An independent Data Safety Monitoring Board has been assembled and reviews study progress, including safety concerns and adverse events, twice per year. All reports are shared with the IRB and the funder at least annually.

### Dissemination plan

In addition to reporting on clinicaltrials.gov, findings from this study will be disseminated via peer-reviewed publications and conference abstracts/presentations. Moreover, presentations at community organizations, including our partner SSP sites, and government entities (e.g., local public health departments, CDC) will be conducted. We will also create easy-to-read infographics to share via social media and our research group website(s).

## Discussion

In the context of rising HIV transmission among PWID nationally, [1–4] improved use of evidence-based HIV prevention strategies is urgently needed. Though PrEP (in the form of daily oral antiretroviral medications) has been approved for use for more than a decade, uptake among PWID has been stagnant and numerous hypothesized PrEP adherence and persistence challenges exist [26, 27]. From our formative research, we learned that interventions needed to address multiple, multilevel barriers to PrEP utilization among PWID, including low HIV knowledge and perceived risk, limited PrEP knowledge and interest, and interpersonal, clinical, and structural barriers to healthcare utilization [26, 27]. Drawing from Social Cognitive Theory (SCT) and evidence on the benefits of patient navigation, we selected individually tailored education, motivational interviewing, problem-solving and planning, and ongoing navigation support as our key intervention strategies. To test the efficacy of the PrEP for Health intervention in improving PrEP uptake, adherence, and persistence among PWID, we designed the RCT described in this paper in close collaboration with our community partner organizations (SSPs) in two communities in MA that have been heavily impacted by opioid and polysubstance use and injection-related HIV transmission. We believe our resulting study design has several key strengths and innovations, including our community-informed approach, our rigorous yet “real-world” study design, and the flexibility of our intervention to evolving modalities, as described below.

First, in addition to selecting intervention strategies and a delivery format supported by behavioral theory and evidence from our formative research, we also received critical input from our community research partner organizations (SSPs). Importantly, we learned that most—if not all—of the theory- and evidence-based strategies we selected were feasible and culturally appropriate within SSP contexts. For example, many SSPs already provide PrEP information and screening services (e.g., HIV testing) that can support the initial stages of the PrEP care continuum. Similarly, we learned that MI is well-aligned with SSPs’ harm reduction-orientation and models of client-centered service delivery. In terms of intervention delivery, we also received community input on the need for primarily in-person research activities. Despite the restrictions imposed by the COVID-19 pandemic on research activities and SSP services, we were aware that a large proportion of our study population lacks consistent phone and Internet access. Thus, to reach a wide swatch of local PWID and ensure that some of the most marginalized individuals (i.e., those completely lacking phone and Internet access) were not excluded, we temporarily delayed study initiation during COVID-19-related closures of our SSP sites to eventually launch an in-person study with appropriate safety measures in place.

Similarly, while we initially considered delivering all intervention content and strategies within a single session, our SSP research partners advised us to split the content across two sessions of more limited length (i.e., ≤ 45 min) to reduce participant burden. Upon implementing the study, we also learned that referrals from SSPs to nearby PrEP prescribers, along with participants’ decisions to initiate PrEP, can often require days to weeks, so our separation of the intervention content into two sessions, spaced approximately one month apart, enabled the first session to focus on PrEP uptake and the second session to either revisit that content or proceed on to PrEP adherence and persistence, depending on participants’ needs. These two sessions, in addition to being more manageable for interventionists and participants, thus enabled more specific attention to key barriers and plans relating distinct steps in the PrEP care continuum. Anecdotally, we have also observed how the scheduling and planning required for the second session promotes continued interaction and rapport between interventionists and participants.

Second, to enhance scalability if PrEP for Health is found to be efficacious, we designed a rigorous yet “real-world” intervention to help maximize widespread implementation. Our study takes place in two distinct sites in terms of inner (i.e., organizational) and outer contextual (i.e., community) factors. While our intervention and assessment procedures are identical across sites, we worked with our SSP research partners to develop recruitment and retention protocols to maximally engage participants in the study; while we use most of our recruitment and retention activities in both sites, differences in the communities (e.g., in terms of urban density, availability of public transportation options) have required regular reconsideration of which strategies to prioritize in each site. By being in constant community with our SSP research partners through regular all-team meetings and daily interactions with study staff, we have also been able to improve our referral processes, for example, by ensuring that staff are present on days when PrEP prescribers are onsite or in nearby mobile clinics. Maintaining flexibility in some aspects of our approach based on our understanding of real-world organizational and community factors (e.g., changing our study schedule to be present on days when mobile clinics are nearby) has supported implementation of the trial so far.

Third, we have remained aware of recent advances in biomedical HIV prevention technologies and were pleased that cabotegravir received U.S. FDA-approval for use as long-acting injectable as PrEP (LAIP). Research by our team and others has found LAIP to be preferred over daily oral PrEP in several PWID samples because it would eliminating the need for adherence to a daily medication, which is particularly challenging for PWID who frequently experience homelessness, forced residential relocation, incarceration, and loss or theft of personal belongings including medications [26, 27, 32, 88, 89]. Despite this important advantage of LAIP, there are still notable barriers to initial uptake (one of our primary outcomes) and hypothesized challenges with PrEP persistence (our secondary outcome). As such, if local prescribers begin prescribing LAIP in the communities where our participants reside as part of their clinical standard of care, it will be available to them, and we will continue to track their PrEP uptake, adherence and persistence outcomes. We have considered how the flexibility of our intervention content, which was designed to be tailored to individuals’ unique needs, can be responsive to evolving PrEP modalities. Furthermore, from all participants, we are collecting data on interest in LAIP (via surveys) which can support future research or programmatic efforts to increase availability of this modality to PWID, who have unfortunately been excluded from much PrEP research to date, including efficacy trials of injectable PrEP [24].

### Limitations

There are potential limitations of the proposed project. First, the RCT may not be powered to determine intervention efficacy for our secondary outcome of PrEP persistence; however, findings may still help inform subsequent research. Second, by situating this study within two urban SSPs in Massachusetts, findings may not generalize to other geographic regions or PWID who do not access SSPs. However, our selected SSP sites cover distinct metropolitan areas and are community-based venues frequented by large numbers of PWID who are diverse in terms of race, ethnicity, age, gender, and sexual orientation, possibly enhancing the generalizability of findings. Moreover, our mediation and moderation analyses will provide more insight into both mechanisms of action and subgroup differences that can inform future broadening in scope. Finally, although this study will not directly address issues of cost-effectiveness of PrEP for PWID, implementation-related findings could inform research on other health interventions for this medically underserved population.

## Conclusion

In summary, based on formative work and community collaboration, we developed the theory-based, multicomponent “PrEP for Health” intervention targeting PrEP knowledge, motivation, self-efficacy, behavioral skills, and structural barriers to access among PWID at risk of HIV acquisition. Importantly, this RCT is based in two SSPs in a region experiencing ongoing HIV transmission associated with injection drug use, making our study sites ideal settings for the evaluation of PrEP interventions for PWID. If our PrEP navigator-delivered behavioral intervention is found to be efficacious in improving PrEP uptake, adherence, and persistence, findings could inform the dissemination of this model to diverse SSPs and possibly other community-based settings. Future research could also investigate whether components of PrEP for Health, if successful, could be adapted to support utilization of HCV treatment, medications for substance use disorders, or other bio-behavioral prevention services for this marginalized population.

## Data Availability

Reasonable requests to access study data will be considered by the corresponding authors(s), and upon completion of planned study outcome analyses, raw data will be freely available to any scientist wishing to use them for non-commercial purposes, without breaching patient confidentiality, per journal guidelines.

## List of abbreviations

API: Attributable Proportion due to Interaction
ELISA: enzyme-linked immunosorbent assay
eSOC: enhanced standard of care
GEE: generalized estimating equation
GLM: generalized linear model
HCV: Hepatitis C Virus
HIV: Human Immunodeficiency Virus
IRB: institutional review board
LAIP: long-acting injectable pre-exposure prophylaxis
MA: Massachusetts
MI: motivational interviewing
MINT: Motivational Interviewing Network of Trainers
NIDA: National Institute on Drug Abuse
PrEP: pre-exposure prophylaxis
PWID: people who inject drugs
RCT: randomized controlled trial
SCT: Social Cognitive Theory
SEM: structural equation modeling
SSP: syringe service program
TAF: tenofovir alafenamide
TDF: tenofovir disoproxil
